# Floret Biofortification of Broccoli Using Amino Acids Coupled with Selenium under Different Surfactants: A Case Study of Cultivating Functional Foods

**DOI:** 10.3390/plants12061272

**Published:** 2023-03-10

**Authors:** Dimitris L. Bouranis, Georgios P. Stylianidis, Vassiliki Manta, Evangelos N. Karousis, Andriani Tzanaki, Despina Dimitriadi, Emmanuel A. Bouzas, Vassilis F. Siyiannis, Violetta Constantinou-Kokotou, Styliani N. Chorianopoulou, Elke Bloem

**Affiliations:** 1 Plant Physiology & Morphology Laboratory, Crop Science Department, Agricultural University of Athens, Iera Odos 75, 118 55 Athens, Greece; 2 PlanTerra Institute for Plant Nutrition & Soil Quality, Agricultural University of Athens, Iera Odos 75, 118 55 Athens, Greece; 3 Karvelas AVEE, 80 km N.R. Athens - Lamia, 32200 Ypato, Greece; 4 Chemical Laboratories, Department of Food Science and Human Nutrition, Agricultural University of Athens, 11855 Athens, Greece; 5 Geoponiki SA, 26.5 km Lavrion Avenue, 194 00 Koropi, Greece; 6 Julius Kuehn Institute, Federal Research Centre for Cultivated Plants, Bundesallee 58, 38116 Braunschweig, Germany

**Keywords:** sulfur, cysteine, methionine, phenylalanine, tryptophane, glucosinolates, polyphenols, selenium, isodecyl alcohol ethoxylated, organosilicon surfactant

## Abstract

Broccoli serves as a functional food because it can accumulate selenium (Se), well-known bioactive amino-acid-derived secondary metabolites, and polyphenols. The chemical and physical properties of Se are very similar to those of sulfur (S), and competition between sulfate and selenate for uptake and assimilation has been demonstrated. Towards an efficient agronomic fortification of broccoli florets, the working questions were whether we could overcome this competition by exogenously applying the S-containing amino acids cysteine (Cys) or/and methionine (Met), or/and the precursors of Glucosinolate (GSL) types along with Se application. Broccoli plants were cultivated in a greenhouse and at the beginning of floret growth, we exogenously applied sodium selenate in the concentration gradient of 0, 0.2, 1.5, and 3.0 mM to study the impact of increased Se concentration on the organic S (S_org_) content of the floret. The Se concentration of 0.2 mM (Se0.2) was coupled with the application of Cys, Met, their combination, or a mixture of phenylalanine, tryptophane, and Met. The application took place through fertigation or foliar application (FA) by adding isodecyl alcohol ethoxylate (IAE) or a silicon ethoxylate (SiE) surfactant. Fresh biomass, dry mass, and Se accumulation in florets were evaluated, along with their contents of S_org_, chlorophylls (Chl), carotenoids (Car), glucoraphanin (GlRa), glucobrassicin (GlBra), glucoiberin (GlIb), and polyphenols (PPs), for the biofortification efficiency of the three application modes. From the studied selenium concentration gradient, the foliar application of 0.2 mM Se using silicon ethoxylate (SiE) as a surfactant provided the lowest commercially acceptable Se content in florets (239 μg or 0.3 μmol g^−1^ DM); it reduced S_org_ (−45%), GlIb (−31%), and GlBr (−27%); and it increased Car (21%) and GlRa (27%). Coupled with amino acids, 0.2 mM Se provided commercially acceptable Se contents per floret only via foliar application. From the studied combinations, that of Met,Se0.2/FA,IAE provided the lowest Se content per floret (183 μg or 0.2 μmol g^−1^ DM) and increased S_org_ (35%), Car (45%), and total Chl (27%), with no effect on PPs or GSLs. Cys,Met,Se0.2/FA,IAE and amino acid mix,Se0.2/FA,IAE increased S_org_ content, too, by 36% and 16%, respectively. Thus, the foliar application with the IAE surfactant was able to increase S_org_, and methionine was the amino acid in common in these treatments, with varying positive effects on carotenoids and chlorophylls. Only the Cys,Met,Se0.2 combination presented positive effects on GSLs, especially GlRa, but it reduced the fresh mass of the floret. The foliar application with SiE as a surfactant failed to positively affect the organic S content. However, in all studied combinations of Se 0.2 mM with amino acids, the Se content per floret was commercially acceptable, the yield was not affected, the content of GSLs was increased (especially that of GlRa and GlIb), and PPs were not affected. The content of GlBr decreased except for the treatment with methionine (Met,Se0.2/FA,SiE) where GlBr remained unaffected. Hence, the combination of Se with the used amino acids and surfactants can provide enhanced biofortification efficiency in broccoli by providing florets as functional foods with enhanced functional properties.

## 1. Introduction

Functional foods of plant origin are composed of naturally occurring components in increased concentrations compared with traditional food [1]. Fortification and biofortification are both food enrichment technologies; however, they differ in their approach. In fortification technology, the fortificants are added directly to the food during processing, after its production. Biofortification technology involves fortification at the cultivation level of the food; it is a process of fortifying crops naturally during their growth before the processing. The available approaches for biofortifying crops are through agronomic practices, breeding, or biotechnology [2]. Broccoli (*Brassica oleracea* var. *italica*) serves as a functional food, as it offers many health-promoting properties because it contains antioxidant and anticarcinogenic compounds consumed for their potential health-promoting properties. The health benefits of broccoli among others are associated with bioactive secondary plant metabolites [3]. Among these compounds, glucosinolates (GSLs), sulforaphane, and polyphenols (PPs) are of major interest [4,5]. Moreover, broccoli can accumulate selenium (Se), and Se metabolites are also known for their potential in cancer prevention [6]. Agronomic biofortification is a prevalent strategy for battling human Se deficiency by enriching crops with Se to secure its adequate supply [7,8].

Selenium is not an essential element for plants; however, it is an essential element for animals and humans, and it is acquired largely from plants [9,10]. Selenium enters the diet primarily through the ingestion of plant and/or animal products. In animals and humans, Se acts as an antioxidant and supports reproduction, immune responses, and thyroid hormone metabolism [11]. Selenium deficiency affects hundreds of millions of people worldwide, particularly in developing countries, and there is increasing awareness that a suboptimal supply of Se can also negatively affect human health [6]. The recommended intake for a person is 55 μg day^−1^, and the maximum safe dietary intake is in the range of 400 μg day^−1^ [12,13]. Τhe German, Austrian, and Swiss nutrition societies have revised the reference values for the intake of selenium to 70 µg day^−1^ for men and 60 µg day^−1^ for women [14]. Se fertilization of broccoli crops results in a significant increase in Se concentration in the vegetable [15]. The Se content in soil is usually low and the fertilization of broccoli crops with Se is a way to enrich this vegetable with Se metabolites [16]. The Se concentration in edible plants is determined by the Se phytoavailability in soils, and it can be toxic above a certain soil level as well. Most plants that grow on seleniferous soils which accumulate <100 μg Se g^–1^DM cannot tolerate greater tissue Se concentrations. Some plant species are Se accumulators as they can accumulate tissue Se concentrations >100 μg Se g^–1^DM. Some species, called Se hyperaccumulators, can even accumulate Se concentrations of 1000–15,000 μg Se g^–1^ DM [10]. Se at low doses protects plants from a variety of abiotic stresses (drought, cold, desiccation, and metal stress). At high doses, Se induces oxidative stress and distorts protein structure and function, which is the main cause of Se toxicity in plants [17,18,19]. As Se deficiency in the human diet is a global problem, plants that accumulate Se represent a major source of Se to consumers [7,20].

Apart from its outstanding capacity to accumulate Se, broccoli has gained great attention because of its high content of GSLs and phenolic compounds [16]. Glucosinolates are sulfur (S)- and nitrogen (N)- containing compounds that are composed of a sulfonated oxime group, linked to a thioglucose group, and an amino-acid-derived side chain [6,21]. Moreover, GSLs are amino-acid-derived natural compounds that are divided into three groups: aliphatic, phenyl (or aromatic), and indolic GSLs, depending on their amino acid precursors and structure. Aliphatic GSLs are derived from methionine, alanine, leucine, isoleucine, valine, or glutamate. Phenyl GLSs are synthesized from phenylalanine and tyrosine, whilst indole GSLs are made from tryptophan [22]. Plants of the Brassicales order constitutively allocate carbon (C), N, and S to synthesize GSLs as their primary defense metabolites. Exogenous C sources have general quantitative effects on GSL accumulation, whilst S or N limitation results in distinct changes in GSL profiles, indicating that these macronutrients provide different regulatory inputs [23]. While intact GSLs are biologically inactive, various products, including isothiocyanates, nitriles, epithionitriles, and cyanides obtained through the hydrolysis of GSLs, exhibit many different biological activities, among which several therapeutic benefits have been suggested [24]. In particular, glucoraphanin, the major aliphatic GSL found in broccoli, is hydrolyzed by myrosinase to yield sulforaphane, an isothiocyanate that exhibits high anticarcinogenic activity [25].

Polyphenols are another target for biofortification because they are abundant constituents in our diet, and evidence for their role in the prevention of degenerative diseases such as cancer and cardiovascular diseases is emerging [26]. Broccoli is a good source of health-promoting compounds since it also contains PPs [5]. Polyphenols are secondary compounds widely distributed in the plant kingdom. They are divided into several classes, i.e., phenolic acids, flavonoids, stilbenes, and lignans, which are distributed in plants and food of plant origin [27]. Polyphenols are the most abundant antioxidants in the diet. Their total dietary intake could be as high as 1 g day**^−^**^1^, which is much higher than that of all other classes of phytochemicals and known dietary antioxidants [28]. The best way to prevent several diseases is the consumption of an optimal diet containing natural antioxidants. There is abundant proof that PPs are efficient antioxidants, stronger than antioxidant vitamins. Within the bioactive compounds of broccoli, PPs seem to have the greatest effect [29].

The dynamically changing environmental conditions promote a complex regulation of plant metabolism as well as balanced resource investments to development and defense. As plants represent the main dietary source of Se, Se-biofortified crops may be used as a means to deliver Se to consumers. However, selenium biofortification programs must consider the interactions between Se and the main metabolic pathways of the plant [30,31,32]. Se assimilation in plants affects both S and N metabolic pathways. According to Malagoli et al. (2015) [30], this is the reason why recent research has also focused on the effect of Se fertilization on the production of S- and N-secondary metabolites with putative health benefits. Selenium is chemically analogous to S and is accumulated by all plants to some extent, in all plant parts. The chemical and physical properties of Se are very similar to those of S, as summarized by Whanger (2004) [12]. Briefly, the two elements have similar outer valence shell electronic configurations and atomic sizes, and their bond energies, ionization potentials, and electron affinities are virtually the same. On the other hand, however, the biochemistry of Se and S differs in at least two respects that distinguish them in biological systems. Firstly, in biological systems, selenium compounds are metabolized to more reduced states, whereas S compounds are metabolized to more oxidized states. Secondly, moreover, the chemical behavior of these elements is different, especially in the acid strengths of their hydrides H_2_S and H_2_Se [12]. The Se levels found in crops depend not only on soil Se abundance but also on the levels of competing S compounds [7]. Care must be taken not to supply unnecessary S in Se-fortified crop production since S will reduce Se uptake [30]. Primary S assimilation is a prerequisite for synthesizing GSLs in Brassicales. GSL accumulation is responding to plant development and abiotic factors, such as N and S supply [33]. The backbone of GSLs contains three S atoms, which can account for up to 30% of the total sulfur content of the entire plant [34].

In the agronomic biofortification process, the application may take place through fertigation via the roots or foliarly. Fertilization characteristics including formulation, dose, and timing were found to be driving variables enhancing crop Se uptake. The highest uptake efficiencies were found for foliar Se-based fertilizers [35,36,37]. Usually, in foliar applications, a wetter or surfactant (surface active agent) is incorporated. Surfactants are commonly incorporated into agrochemical formulations to enhance the biological efficiency of foliar sprays by improving the wetting behavior of the spray and/or the penetration of the active ingredients into the leaf tissues. Penetration-accelerating surfactants are known to increase the cuticular permeability and may submit the cuticular barrier to water loss [38]. Among the various types of surfactants, two are in common use in the experimental area: isodecyl alcohol ethoxylate (IAE) surfactants or organosilicon-based ethoxylate (SiE) ones. Ethoxylated surfactants may improve spray retention and leaf wetting, whilst they may also increase cuticular permeability [39]. Alcohol ethoxylates are used as surfactants in a wide variety of agrochemical formulations to enhance the effectiveness of the active constituents. Alcohol ethoxylates belong to the class of compounds which are synthesized via the reaction of a fatty alcohol and ethylene oxide, resulting in a molecule that consists of two parts: one a carbon-rich, fatty alcohol and the second part a hydrophilic polyoxyethylene chain [40]. As regards SiE, the silicon–oxygen bonds are hydrophobic, whilst the ethoxylated clusters are hydrophilic, creating a wetting agent that spreads quickly, thus covering a large surface area, greater than conventional surfactants. Silicone surfactants undergo a relatively rapid hydrolytic cleavage in the environment, as do linear silicone polymers, to give monomers that are more slowly converted by oxidation back to water, CO_2_, and sand [41,42]. Generally, surfactant effects are species- and compound-specific [43].

As the impact of Se biofortification on the S content of the broccoli florets raises a major point in the agronomic handling of broccoli crop, it was the target of this work to optimize the fertilization strategy. In the first part of this case study, we focused on the effect of a gradient of Se concentrations to quantify the effect of Se concentration on organic S (S_org_), GSL, and PP content and to select an optimum Se concentration for further trials (first working target). The second working target was whether we could overcome the negative impact of the Se application on S uptake and utilization by exogenously applying the S-containing amino acids cysteine or/and methionine. The third working approach was whether we could biofortify the Se-treated florets by applying the precursors of glucosinolate types, and the various provided AA combinations were coupled with the selected Se concentration. Among the various types of surfactants, two are in common use in the experimental area: IAE or SiE ones, and in a commercial application, they are among the first choice to be used by the farmer for foliar application. Hence, the fourth working target was whether the type of the surfactant affects the efficiency of the applied Se concentration, coupled or not with the S-containing amino-acid-based biofortification.

## 2. Results

To this end, 150 broccoli plants (cv. Sonora) were cultivated in a greenhouse, where we compared the application of 0, 0.2, 1.5, and 3.0 mM Se, applied once and at the beginning of the floret growth. The Se 0.2 mM level was combined with cysteine (0.05 mM), and/or methionine (0.1 mM), or with the mixture of phenylalanine (0.25 mM), tryptophane (0.05 mM), and methionine (0.1 mM). Three application modes were studied: fertigation (FERT), compared with foliar application assisted by IAE- or SiE-based surfactant (FA,IAE vs. FA,SiE). The efficiency of floret biofortification was assessed and evaluated in terms of fresh mass (FM) and dry mass (DM), and the contents in florets of Se, total S (S_tot_), sulfate (SO4-S), organic S (S_org_), carotenoids (Car), total chlorophylls (Chltot), chlorophyll-a (Chl*a*), chlorophyll-b (Chl*b*), total polyphenols (PP), the sum of the determined glucosinolates (ΣGSL), glucoraphanin (GlRa), glucoiberin GlIb), and glucobrassicin (GlBr).

### 2.1. The Performance of the Treatments and the Application Modes with Increased Selenium Concentration on the Biofortification of Florets

*Fresh mass*—The FM of the broccoli florets of the control treatment (Se 0.0/FERT) was 77 ± 4 g. Among the treatments, the FM was affected, whilst among the application modes, it was not. From the treatments, Se 0.2 presented the highest values Table 1 (1.A). The applications that reduced the FM were Se1.5/FERT (−17%) and Se3.0/FERT (−30%), Se0.0/IAE (−21%), Se0.0/SiE (−21%), Se 3.0/SiE (−16%), and Se1.5/SiE (−21%) (Table 4).

*Dry mass*—The DM of the florets of the control treatment was 11 ± 0.9 g. Statistically, the DM of the florets was not affected, neither by the treatment nor by the application mode Table 1 (2.A). The treatments that presented a tendency to reduce the DM were Se0.0/FA,IAE (−24%) and Se1.5 (−15%) (Table 4).

*Selenium*—The data of the Se contents of all three treatments and application modes revealed significant differences between treatments and Se application with the highest value found for Se3.0/FERT (lowercase letters). If the Se application is compared (TR) independently of the application mode, Se3.0 revealed the significantly highest Se content (uppercase letter: A). On the other hand, if the application mode (AppM) is compared, FERT resulted in the significantly highest Se content (A’). Relative to Se0.2/FERT, the applications Se3.0/FERT and Se1.5/FERT increased the Se content by 8.5 and 5.6 times. The foliar application of Se reduced the Se content of the floret, with FA,SiE presenting a tendency towards the lowest contents in Table 1 (3.A). Relative to Se0.2/FERT, Se3.0/FA,IAE, Se1.5/FA,IAE, and Se0.2/FA,IAE increased the Se content by 5.3, 2.8, and 0.8 times, respectively, whilst Se3.0/FA,SiE, Se1.5/FA,SiE, and Se0.2/FA,SiE increased the Se content by 4.6, 3.2, and 0.4 times, respectively (Table 4).

*Total sulfur*—The S_tot_ content of the control florets was 60.2 ± 6.1 μmol g^−1^ DM. Se 3.0/FERT reduced the S_tot_ by −23%. The Se 0.0/IAE and Se 3.0/IAE treatments decreased S_tot_ by 22% and 30%, respectively. Negative results were achieved in the cases of Se 3.0/SiE (−23%) and Se 0.2/SiE (−35%) (Table 1 (4.A), Table 4).

*Sulfate*—The SO4-S content of the control florets was 11.3 ± 0.7 μmol g^−1^ DM. In almost all treatments and application modes, the SO4-S content was found to be increased. As regards fertigation, the increase in SO4-S content was as follows: Se3.0/FERT, 65%; Se1.5/FERT, 68%; and Se0.2/FERT, 59%. The same picture was provided by the foliar application with IAE: Se3.0/FA,IAE, 128%; Se1.5/FA,IAE, 117%; and Se0.2/FA,IAE, 84%. In the foliar application mode with SiE, Se0.2/FA,SiE did not increase SO4-S. The other Se treatments increased the SO4-S content: Se3.0/FA,SiE by 69% and Se1.5/FA,SiE by 87%. (Table 1 (5.A), Table 4).

*Organic sulfur*—The S_org_ content of the control florets was 48.9 ± 5.6 μmol g^−1^ DM. The application of Se by fertigation reduced the S_org_ content by 43% and 25% in the cases of Se3.0/FERT and Se0.2/FERT, respectively. The following applications reduced the S_org_ content: Se0.0/FA,IAE (−25%), Se3.0/FA,IAE (−67%), Se1.5/FA,IAE (−34%), Se0.2/FA,IAE (−20%), Se3.0 /FA,IAE (−44%), Se1.5/FA,IAE (−33%), and S 0.2/FA,IAE (−45%) (Table 1 (6.A), Table 4).

*Carotenoids*—The Car content of the control florets was 0.14 ± 0.02 mg g^−1^ DM. Statistically, neither the treatment nor the application mode affected the Car content. Se0.2/FERT presented a tendency to decrease it by 21%, whilst Se0.0/FA,IAE as well as Se0.2/FA,SiE presented a tendency to increase the Car content by 21% (Table 2 (1.A), Table 4).

*Total chlorophylls*—The Chl_tot_ content of the control florets was 1.44 ± 0.11 mg g^−1^ DM. Statistically, neither the treatment nor the application mode affected the Chl_tot_ content. Se0.2/FERT presented a tendency to decrease it by 17% (Table 2 (2.A), Table 4).

*Chlorophyll-a*—The Chl*a* content of the control florets was 0.99 ± 0.08 mg g^−1^ DM. The treatments and/or application modes did not statistically affect the Chl*a* content. Se0.2/FERT presented a tendency to decrease Chl*a* by 19% (Table 2 (3.A), Table 4).

*Chlorophyll-b*—The Chl*b* content of the control florets was 0.44 ± 0.02 mg g^−1^ DM. Neither the Se concentration nor the application mode statistically affected the Chl*b* content (Table 2 (4.A), Table 4).

*Polyphenols*—Within the treatments, the application mode presented differentiations, with Se0.2 presenting higher contents. Within application modes, FA,SiE presented the lowest contents. Se3.0/FERT, Se1.5/FERT, and Se0.2/FER increased the PP content by 18%, 15%, and 19%, respectively. The treatments Se3.0/FA,IAE, Se1.5 /FA,IAE, and Se0.2/FA,IAE increased the content by 21%, 12%, and 28%, respectively (Table 3 (1.A), Table 4).

*The sum of determined glucosinolates*—The ΣGSL content of the control florets was 2.34 ± 0.18 mg g^−1^ DM. Within the application modes, the treatments presented differentiations. Se3.0/FERT and Se1.5/FERT decreased ΣGSL by 19% and 17%, respectively, whilst Se0.2/FERT increased ΣGSL by 21%. Se0.0/FA,IAE increased the ΣGSL content by 21%. Moreover, FA,IAE affected the ΣGSL content in all Se treatments: Se3.0 / FA,IAE by 38%; Se1.5/FA,IAE by 44%; and Se0.2/FA,IAE by 24%. Se3.0/FA,SiE and Se1.5/FA,SiE increased the ΣGSL content by 17% and 30%, respectively (Table 3 (2.A), Table 4).

*Glucoraphanin*—The GlRa content of the control florets was 1.64 ± 0.14 mg g^−1^ DM. Within the application modes, GlRa content presented differentiations. Se0.2/FERT presented a tendency to increase the content by 32%. Se0.0/FA,IAE increased the GlRa content by 35%, whilst the Se3.0/FA,IAE, Se1.5/FA,IAE, and Se0.2/FA,IAE treatments increased the GlRa content by 54%, 58%, and 26%, respectively. Se3.0/FA,SiE, Se1.5/FA,SiE, and Se0.2/FA,SiE increased the GlRa content by 38%, 55%, and 27%, respectively (Table 3 (3.A), Table 4).

*Glucoiberin*—The GlIbe content of the control florets was 0.13 ± 0.01 mg g^−1^ DM. The Se 3.0/FERT treatment decreased the content by 15%, whilst Se0.2/FERT increased the content by 38%. Se3.0/FA,IAE, Se1.5/FA,IAE, and Se0.2/FA,IAE increased the content by 62%, 54%, and 69%, respectively. The Se3.0/FA,SiE treatment decreased the content by 54%, Se1.5/FA,SiE increased the content by 15%, whilst Se0.2/FA,SiE decreased the content by 31% (Table 3 (4.A), Table 4).

*Glucobrassicin*—The GlBr content of the control florets was 0.56 ± 0.07 mg g^−1^ DM. Se3.0/FERT and Se1.5/FERT decreased the GlBr content by 32% and 36%, respectively. Se0.0/Fa,IAE decreased the content by 16%. The Se gradient did not affect the GlBr content. Se3.0/FA,SiE, Se1.5/FA,SiE, and Se0.2/FA,SiE decreased the GlBr content by 29%, 36%, and 27%, respectively (Table 3 (5.A) Table 4).

### 2.2. The Performance of the Application Modes and the Treatments with Selenium 0.2 mM Enriched with Amino Acids in the Biofortification of Florets

*Fresh mass*—The FM of the broccoli florets of the control treatment (Se 0.2/FERT) was 81 ± 5 g. The FM of the floret was reduced by Cys,Met,Se0.2/FA,IAE (−22%) and mix,Se0.2/FA,IAE (−23) (Table 1 (1.B), Table 5).

*Dry mass*—The DM of the florets of control treatment was 10.75 ± 0.48 g. The DM was increased by Cys,Se0.2/FERT (24%), whilst Cys,Met,Se0.2/FERT reduced the DM (−16%). All other treatments did not statistically affect the DM. CysSe0.2/FA,IAE increased the DM (17%), whilst Cys,Met,Se0.2/FA,IAE decreased DM (−20%) (Table 1 (2.B), Table 5).

*Selenium*—Cys,Se0.2/FERT, Met,Se0.2/FERT, Cys,Met,Se0.2/FERT, and mix,Se0.2 /FERT increased the Se content by 1.4, 1.1, 1.1, and 1.0 times relative to Se0.2/FERT. In the foliar application with IAE, Cys,Se0.2/FA,IAE, Met,Se0.2/FA,IAE, Cys,Met,Se0.2/FA,IAE, and mix,Se0.2/FA,IAE increased the Se content by 0.5, 0.3, 0.2, and 0.3 times relative to Se0.2/FA,IAE. In the foliar application with SiE, Cys,Se0.2/FA,SiE, Met,Se0.2/FA,SiE, Cys,Met,Se0.2/FA,SiE, and mix,Se0.2/FA,SiE increased the Se content by 0.5, 0.3, 0.5, and 0.5 relative to Se 0.2/FA,SiE (Table 1 (3.B), Table 5).

*Total sulfur*—The S_tot_ content of the control florets was 54.5 ± 3.3 μmol g^−1^ DM. Cys,Met,Se0.2/FERT decreased S_tot_ (−24%). In the foliar application with IAE, S_tot_ was increased by Met,Se0.2/FA,IAE (35%) and Cys,Met,Se0.2/FA,IAE (34%). In the foliar application with SiE, negative results were provided in all treatments: Cys,Se0.2/FA,SiE (−28%), Met,Se0.2/FA,SiE (−36%), Cys,Met,Se0.2/FA,SiE (−24%), and mix,Se0.2/FA,SiE (−17%). Considering the overall behavior of the application modes, the IAE surfactant showed higher values of S_tot_ content than the SiE surfactant and fertigation (Table 1 (4.B), Table 5).

Sulfate—The SO4-S content of the control florets was 18.0 ± 1.6 μmol g^−1^ DM. The SO4-S content was found increased in the following treatments: Cys,Met,Se0.2/FERT (53%), mix,Se0.2/FERT (20%), Cys,Met,Se0.2/FA,IAE (36%), mix,Se0.2/FA,IAE (31%), and Se0.2/FA,SiE (29%) (Table 1 (5.B), Table 5).

*Organic S*—The S_org_ content of the control florets was 36.5 ± 1.7 μmol g^−1^ DM. In the fertigation mode, Cys,Met,Se0.2/FERT reduced S_org_ (−62%). In the FA,IAE mode, S_org_ was found increased in Cys,Se0.2/FA,IAE (27%), Met,Se0.2/FA,IAE (55%), and Cys,Met,Se0.2/FA,IAE (33%). In the FA,SiE mode, all combinations reduced S_org_: Cys,Se0.2/FA,SiE (−52%), Met,Se0.2/FA,SiE (-%), Cys,Met,Se0.2/FA,SiE (−32%), and mix,Se0.2/FA,SiE (−41%) (Table 1 (6.B), Table 5).

*Carotenoids*—The Car content of the control florets was 0.11 ± 0.01 mg g^−1^ DM. All treatments increased the Car content, regardless of the application mode (Table 2 (1.B), Table 5).

*Total chlorophylls*—The Chl_tot_ content of the control florets was 1.19 ± 0.07 mg g^−1^ DM. Compared with the Se0.2/FERT, almost all combinations of Se0.2 with AA presented a tendency to increase Chl_tot_. The only exception was the Cys,Se0.2/FA,IAE with no effect on Chl_tot_ content (Table 2 (2.B), Table 5).

*Chlorophyll-a*—The Chl*a* content of the control florets was 0.80 ± 0.06 mg g^−1^ DM. The same pattern as that of Ch_ltot_ was observed for the Chl*a* contents (Table 2 (3.B), Table 5).

*Chlorophyll-b*—The Chl*b* content of the control florets was 0.38 ± 0.01 mg g^−1^ DM. The same pattern as that of Chl_tot_ was observed for the Chl*b* contents, too (Table 2 (4.B), Table 5).

*Polyphenols*—The PP content of the control florets was 9.87 ± 0.28 mg g^−1^ DM. The combination of Se0.2 and AA did not affect the PP content in all application modes (Table 3 (1.B), Table 5).

*The sum of determined glucosinolates*—The ΣGSL content of the control florets was 2.83 ± 0.18 mg g^−1^ DM. In Cys,Se0.2/FERT, the fertigation mode did not affect the ΣGSL content. In contrast, it was increased in the following treatments: Met,Se0.2/FERT (27%), Cys,Met,Se0.2/FERT (37%), and mix,Se0.2/FERT (38%). In the FA,IAE mode, Cys,Met,Se0.2/FA,IAE was positively affected (37%). In the FA,SiE mode, all combinations with AA were positively affected: Cys,Se0.2/FA,SiE (42%), Met,Se0.2/FA,SiE (50%), Cys,Met,Se0.2/FA,SiE (54%), and mix,Se0.2/FA,SiE (52%). In general, the SiE surfactant achieved higher values of ΣGSL than the IAE surfactant and fertigation mode (Table 3 (2.B), Table 5).

*Glucoraphanine*—The GlRa content of the control florets was 2.16 ± 0.15 mg g^−1^ DM. In all application modes, Se0.2 mM coupled with the selected AA increased the GlRa content. In the fertigation mode, the following increases were observed: Met,Se0.2/FERT (36%), Cys,Met,Se0.2/FERT (42%), and mix,Se0.2/FERT (45%). In the foliar application with IAE only, Cys,Met,Se0.2/FA,IAE increased the GlRa content (54%). In the foliar application with SiE, an increase was provided by Cys,Se0.2/FA,SiE (49%), Met,Se0.2/FA,SiE (64%), Cys,Met,Se0.2/FA,SiE (68%), and mix,Se0.2/FA,SiE (64%) (Table 3 (3.B), Table 5).

*Glucoiberin*—The GlIbe content of the control florets was 0.18 ± 0.00 mg g^−1^ DM. In the fertigation mode, the GlIb content was increased in almost all combinations with Se0.2 mM: Met,Se0.2/FERT (39%), Cys,Met,Se0.2/FERT (33%), and Mix,Se0.2/FERT (33%). Foliar application with mix,Se0.2/FA,IAE negatively affected the content (−22%). In the foliar application mode with SiE, Se0.2/FA,SiE decreased the content (−50%). In contrast, all combinations of AA with Se0.2 mM increased the content: Cys,Se0.2/FA,SiE (56%), Met,Se0.2/FA,SiE (61%), Cys,Met,Se0.2/FA,SiE (78%), and mix,Se0.2/FA,SiE 2 (117%) (Table 3 (4.B), Table 5).

*Glucobrassicin*—The GlBr content of the control florets was 0.49 ± 0.04 mg g^−1^ DM. In the fertigation mode, negative effects were found for Cys,Se0.2/FERT (−31%). The foliar application mode with IAE negatively affected Cys,Met,Se0.2/FA,IAE (−20%), whilst Se0.2/FA,IAE responded positively (27%). The foliar application mode with SiE showed negative effects in the Cys,Met,Se0.2/FA,SiE (−18%) and mix,Se0.2/FA,SiE (−29%) (Table 3 (5.B), Table 5).

## 3. Discussion

### 3.1. Selenium Biofortification and S Nutrition of the Broccoli Floret

Selenium shows chemical similarities to S, and there is a Se-S crosstalk. Selenate is predicted to be taken up by root plasma membrane sulfate transporters [11]. Following assimilation, Se is converted to selenite, and combined with OAS by cysteine synthase to form selenoamino acids, selenocysteine (SeCys), and selenomethionine (SeMet) [10]. SeCys and SeMet can be integrated into proteins to synthesize seleniated proteins [44]. Given that Se is an analog of S and can be toxic to plants, its effect on plant growth is expected to be affected by S nutrition.

To study Se toxicity on broccoli florets, in our preliminary experiments [45], broccoli plants were fortified three times with Na_2_SeO_4_, at the beginning, in the middle, and at the end of floret growth. Plants (cv. Sonora) were grown hydroponically in a greenhouse for 12 weeks, with harvesting taking place at commercial maturity. The plants were treated with two different concentrations of sodium selenate (1.5 mM and 3.0 mM) in the presence and absence of sodium sulfate, and they were divided into six main groups depending on the concentration of Na_2_SeO_4_. The plants were fortified between the 5th and 10th week. Enhanced Se toxicity was observed in the absence of S and resulted in a weight reduction of up to 65%. The amount of water contained in the leaves and florets was the same regardless of Se and S presence. The distribution of Se followed the order: florets > roots > leaves and increased Se application resulted in an increase in Se uptake, particularly in the absence of S. Significant changes were observed in aliphatic GSL hydrolysis product content and only indole-type products were identified [45]. Sulfur deprivation alone did not affect the florets. Selenium 1.5 mM reduced organic S and increased chlorophylls, whilst combined S deprivation coupled with Se 1.5 mM decreased organic S and carotenoids, with no effect on chlorophylls. Selenium 3 mM reduced DM and organic S, whilst combined S deprivation and Se 3 mM reduced organic S, carotenoids, and chlorophylls. Due to the enhanced Se content, the florets were not commercially acceptable [46]. Based on these results, we concluded that the concentration of Se 0.2 mM applied once and at the beginning of the floret growth might provide commercially acceptable results. During the progress of the preliminary experiment, we were monitoring the crop before and after the biofortification for potential transient stressful periods by measuring the optical properties of the leaves, and no visual symptoms were recorded [47,48,49].

Tian et al. (2017) [50] reported an evaluation of the influence of Se treatments on broccoli growth when S was withheld from the growth nutrient solution. They found that Se was highly toxic to plants when S nutrition was poor. In contrast to Se treatments with adequate S nutrition that slightly reduced broccoli growth, the same concentration of Se treatments without S supplementation dramatically reduced plant sizes. Higher Se toxicity was observed with selenate than selenite under low S nutrition. Se toxicity could be counteracted with the increased supplementation of S, and they suggested that this phenomenon is through decreasing the nonspecific integration of Se into proteins and altering the redox system. It was concluded that adequate S nutrition is important to prevent Se toxicity during the biofortification of crops with Se fertilization [50].

In this case study, the application took place once at the beginning of the growth period, i.e., when the florets were 1–2 cm in width. In the first part, we presented the effect of the increasing concentrations of Se on the florets, aiming towards an effective biofortification of florets on a commercial basis. Expressing the results as μg per floret (Table 4), we observed that the Se concentrations of 3.0 mM, 1.5 mM, and 0.2 mM applied by fertigation provided high accumulations, 5.7, 4.4, and 0.7 μmol g^−1^DM, at the level of 5089, 3356, and 597 μg per floret, respectively. In contrast, both foliar applications provided lower Se accumulations. According to Whanger (2004) [9], the maximum safe dietary intake is in the range of 400 μg day^−1^. Only the concentration of Se 0.2 mM, when applied foliarly with the SiE surfactant, provided a Se content of 0.3 μmol g^−1^DM or below the maximum safe value when expressed per floret, i.e., 239 μg per floret. Therefore, Table 4 shows that Se 0.2 mM provided an acceptable dietary intake when foliar application with SiE as the surfactant took place. However, the Se0.2/FA,SiE was found to decrease the S_org_ by 45%, GlIb by 31%, and GlRb by 27%, whilst it tended to increase the contents of Car by 21% and GlRa by 27%. It is remarkable that the Se 0.2 mM treatment presented the highest FM in all the application modes compared with the control and the other studied Se concentrations.

### 3.2. Could We Overcome the Se-S Competition by Exogenously Applying the S-Containing Amino Acids Cysteine or/and Methionine?

According to Godoy et al. (2021) [51], a nongenetic approach to improve crop yield in stress conditions involves the exogenous application of natural compounds, including plant metabolites. In our case, the stress is the contradiction between S and Se uptake and utilization within the floret. Hence, we attempted to overcome this negative impact of Se on S by exogenously applying the S-containing amino acids cysteine or/and methionine in combination with the Se 0.2 mM level. The results showed that in the case of IAE-based foliar application, the Se accumulation was within the range of 0.2–0.3 μmol g^−1^DM, or 136–298 μg per floret, whilst in the case of SiE-based foliar application, it was within the range of 0.2–0.4 μmol g^−1^DM, or 156–313 μg per floret, respectively (Table 5). In the fertigation mode, the Se accumulation was within the range of 0.7–0.8 μmol g^−1^DM, or 568–846 μg per floret. It is worth highlighting that Cys,Se0.2 provided the highest Se accumulation within each application mode, 846 μg per floret in the FERT mode, and 298 and 313 μg per floret in the FA,IAE and FA,SiE modes, respectively.

Expanding the working question, we also attempted to further fortify the Se-treated florets by applying the amino acid precursors of glucosinolate types, as a mixture of phenylalanine, tryptophane, and methionine, in combination with the concentration of 0.2 mM selenium (mix,Se0.2 treatment). Biofortified food of superior nutritional quality may be created and enriched with Se, as well as several other valuable phytochemicals [7]. Besides the need for an improvement in Se content, new challenges also include concerns about the promotion of phytochemicals in plants using Se biofortification. This is due to the various biological properties employed by these phytochemical components, which thereby may encourage positive effects to human health. The main groups of Se-biofortified plants are in Se-accumulating plants, and broccoli is such a crop [52,53,54]. However, Se-enriched broccoli may contain reduced amounts of chemopreventive GSLs. To investigate the basis by which Se treatment influences GSL levels, Tian et al. (2018) [55] treated two broccoli cultivars with Se. It was found that Se supplementation suppressed the accumulation of total GSLs, particularly glucoraphanin, the direct precursor of a potent anticancer compound, in broccoli florets and leaves. They showed that the suppression was not associated with plant S nutrition. The levels of the GSL precursors methionine and phenylalanine as well as the expression of genes involved in GSL biosynthesis were greatly decreased following Se supplementation. Comparative proteomic analysis identified proteins in multiple metabolic and cellular processes that were greatly affected and detected an enzyme affecting methionine biosynthesis that was reduced in the Se-biofortified broccoli. These results indicate that Se-conferred GSL reduction is associated with negative effects on precursor amino acid biosynthesis and glucosinolate–biosynthetic-gene expression and provide information for a better understanding of glucosinolate accumulation in response to Se supplementation in broccoli [55].

In this work, the following questions and criteria for a successful biofortification were evaluated: (1) the accumulated Se per floret to be within an acceptable range, (2) whether the FM was affected, because a decrease in FM is not commercially acceptable, (3) what was the effect on S_org_, (4) what was the impact on the carotenoids, chlorophylls, polyphenols, and glucosinolates. This case study provided the following answers to the addressed questions (Table 5).

In all combinations of the fertigation mode, (1) the Se content per floret rendered the product commercially nonacceptable. Fertigation (2) did not affect the FM, (3) decreased or did not affect the organic S, and (4) did not affect or increased carotenoid or chlorophyll content, did not affect PPs, decreased or did not affect GlBr, and increased GlRa and GlIb.

In contrast to fertigation, the foliar application with the IAE surfactant in all combinations provided florets with an Se content per floret that rendered it commercially acceptable. The Se per floret ranged between 136 and 298 μg, whilst Se0.2/FA,IAE provided 492 μg per floret. The combination of Met,Se0.2/FA,IAE accumulated 183 μg of Se per floret, did not affect FM or DM, increased the organic S (35%) content, increased carotenoid (45%) and chlorophyll (27%) content, and did not affect the PP, ΣGSL, GlRa, GlIb, and GlBr contents. This combination met the criteria for a successful biofortification, and methionine coupled with IAE in a foliar application overcame the Se vs. S competition. On the other hand, the combination of mix,Se0.2/FA,IAE did not meet the criteria: it decreased the FM (by 23%), although it showed a tendency to increase the organic S (16%), and increased chlorophyll and carotenoid content, whilst it did not affect PPs, ΣGSL, GlRa, GlBr, and decreased GlIb (22%). Decreased FM is definitely a disadvantage.

In the foliar application with the SiE surfactant, and in all combinations, the Se content per floret rendered the combinations commercially acceptable. Moreover, it did not affect the FM or DM; however, the S_org_ content was found to be decreased in all combinations. On the contrary, Car, PP, Chl_tot_, ΣGSL, GlRa, GlIb, and GlBr were all increased. Mix,Se0.2/FA,SiE and Met, Se0.2/FA,SiE were the dominant combinations.

### 3.3. The Role of the Surfactant

The aforementioned results clearly show that the used surfactant differentiated the responses. Phenylalanine, tryptophane, and methionine are nonpolar, hydrophobic molecules, whilst cysteine is a polar, uncharged one. The IAE surfactant promoted the increase in the organic S content of the floret in all cases but Se0.2/FA,IAE, which implies that the application mode promoted the utilization of the selected AA, and the finding merits further research. On the other hand, the combination of Cys,Met,Se0.2/FA,IAE promoted the production of more carotenoids, chlorophylls, polyphenols, and glucoraphanins. Coupled with the fact that the combination presented the lowest Se accumulation 136 μg per floret, this is good news indeed, but the FM was decreased by 22%, and this finding merits further research, too.

On the contrary, the foliar application with the SiE surfactant presented decreased organic S in all combinations. With the exception of GlIb (that was found decreased) and PP (that was not affected), carotenoids, chlorophylls, and glucosinolates were found significantly increased. The finding implies that this application mode promoted the production of these compounds. Assuming the accumulation of Se falls within an acceptable range, this application mode might be considered a successful biofortification attempt.

The effects of several organosilicone-type surfactants on the physicochemical properties of spray solutions and on the foliar uptake and field performance of agrochemicals have been reviewed [43]. Is there an effect of Si on S or/and Se? According to Pavlovic et al. (2021) [56], the work on interactions between Si and S or Se is at a preliminary stage. Our results show that the use of the SiE surfactant resulted in a decrease in organic S in all Se concentrations and combinations, which merits further research.

Moreover, adjuvants can also be toxic in their own right, with negative health effects having been reported in humans and on the environment. Adjuvants are regulated differently from active components, with their toxic effects being generally ignored, and are not subject to an acceptable daily intake, nor are they included in the health risk assessment of dietary exposures [57]. To our knowledge, there is no negative reference to the surfactants used in this study; however, because we aim to study the enhanced biofortification of a functional food, such studies need further research, too. Under the circumstances, it would be worthwhile to study decreasing the applied Se concentration to 0.1 mM.

## 4. Materials and Methods

### 4.1. Plant Materials and Cultivation

**Figure 1:**
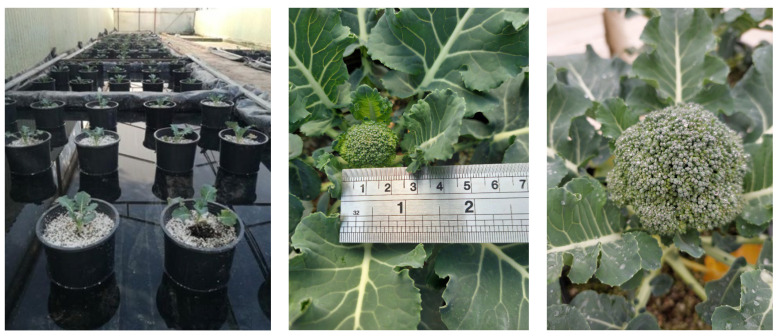
In the left picture are plants after transplantation (stage 14–15 of BBCH scale), along with a broad view of the hydroponics cultivation. In the middle picture is a floret at the beginning of the growth period when the florets were 1–2 cm in width (day 73 post-transplantation, when treatments took place). Each treatment was applied once. In the right is a closer picture of the floret and the leaves. No visual symptoms of any kind were observed during the cultivation period.

The hydroponic cultivation of broccoli took place in a greenhouse (Figure 1). One hundred and fifty broccoli plants of Sonora F1 cultivar *(Brassica oleracea L. var. italica*) were sown on 11 September 2021. On 25 October 2021 (d0), plants that were in the stage of 4^th^–5^th^ unfolded true leaf (stage 14–15 of BBCH scale) were transplanted in 4 L pots (one plant per pot) containing a substrate of sand, perlite, and vermiculite in the ratio 1:1:1, respectively. Then, the pots were placed in 10 different tanks (15 pots per tank) and filled up with water up to a height of 5 cm. Once a week, the plants received a full nutrient solution containing 6 mM KNO_3_, 4 mM Ca(NO_3_)_2_, 2 mM NH_4_H_2_PO_4_, 1 mM MgSO_4_, 0.05 mM KCl, 25 μM H_3_BO_3_, 2 μM MnSO_4_, 2 μM ZnSO_4_, 0.5 μM CuSO_4_, 0.5 μM H_2_MoO_4_, and 0.1 mM NaFeEDTA. The full nutrient solution was applied in a volume of 500 mL per pot, following the cleaning process and the water change in the tanks. The florets appeared on day 60 post-transplantation and the harvest was carried out at day 107, based on market maturity. Plant protection foliar applications for insects and fungi were carried out from the start of the cultivation until d63 post-transplantation.

The treatments took place at d73 post-transplantation. Every treatment consisted of 5 replications. Three different application modes were tested. Fertigation (FERT) was conducted with the application of 100 mL of solution per pot to the substrate. For the two foliar applications, the used surfactants were: a isodecyl alcohol ethoxylate (FA,IAE; Saldo Plus 15 SL) surfactant versus a silicon ethoxylate (FA,SiE; SW7, OMEX Agrifluids LTD) surfactant. Control treatments were prepared for all three applications: the FERT control received no additional application to the weekly fertilization, while FA,IAE and FA,SiE received water containing the corresponding surfactant at the suggested concentration by the company, as a spray application. As biofortification treatments, a series of different solutions were applied, the same in all three application modes. Three different concentrations of sodium selenate (Na_2_SeO_4_) were tested: Se 0.2 mM, Se 1.5 mM, and Se 3 mM. To potentially enhance the organic forms of sulfur, a combination of sodium selenate 0.2 mM and different amino acid solutions were tested in concentrations: methionine 0.1 mM (Met,Se0.2), cysteine 0.05 mM (Cys,Se0.2), and cysteine 0.05 mM + methionine 0.1 mM (Cys,Met,Se0.2), based on previous preliminary experiments.

To potentially enhance the glucosinolate content of the broccoli florets, a mix of three different amino acids was evaluated, consisting of phenylalanine 0.25 mM, methionine 0.1 mM, and tryptophane 0.05 mM, which are precursor molecules for the biosynthesis of aromatic, aliphatic, and indolic glucosinolates, respectively. The mix solution was tested in combination with 0.2 mM sodium selenate (Phe,Trp,Met,Se0.2). All amino acids were fermentation products (CJ Biotech Co., Ltd.—Shenbei New District, Shenyang, Liaoning, China).

### 4.2. Sample Handling and Determinations

At d107 post-transplantation, the harvest of the florets took place. For the separation point of the floret, the last strongly attached leaf on the floret was used as a criterion. After their separation, the florets were kept on a freezer at −18 °C until their lyophilization process. The FM of the florets was measured directly after the sampling, whilst the DM determination took place after the lyophilization process. The frozen florets were lyophilized (using a Lyovapor L-200 lyophilizer, Buchi AG—Flawil, Switzerland); the lyophilized material was ground with a mortar and pestle and then with a RS 200 vibratory disc mill (Retsch GmbH—Haan, Germany) at 1400 rpm for 4 s prior to chemical analyses.

For the determination of the total Se content, a hydride generation–atomic absorption spectrometry (HG-AAS) method was used, based on the combination of previous methods with slight modifications [58,59]. For the sample preparation, 300 mg of the lyophilized sample material was measured and a digestion stage took place with 10 mL of HΝO_3_ 65% at 600 °C for 15 min. Additionally, during the digestion, H_2_O_2_ 30% solution was poured until the solution became transparent. A filtration followed and then a prereduction stage was carried out with 10 mL of HCl 30% in a water bath at 85 °C for 30 min. The final solutions were transferred in 100 mL volumetric flasks and filled up with ultrapure water. The same process was followed for the calibration curve with a 1000 ppm selenium solution (Chem—Lab Analytical B.V.B.A.—Zedelgem, Belgium) in which different dilutions and a blank were further made. The Se content was quantified using a VGA 77 AA hydride generator (Agilent Technologies Inc.—Santa Clara, CA, USA) and an Agilent 240 FSAA Spectrometer (Agilent Technologies Inc.—Santa Clara, CA, USA). For the hydride generation, a 0.6 w/v NaBH_4_ in 0.5 w/v NaOH and a 10 M HCl solutions were used and then the absorbance of the calibration curve and sample solutions was measured.

A turbidimetric method based on the extraction of sulfate anions from acetic acid and their precipitation with boron was used [60,61]. Approximately, 100 mg of the lyophilized sample was extracted for 15 min with 5 mL of CH_3_COOH 2% v/v and after filtering, the extract was supplemented with acetic acid 2% *v*/*v* up to the final volume of 5 mL. The precipitation was carried out by pouring 2000 μL of deionized water, 1000 μL of 500 mM HCl, 1000 μL of Ba—PEG solution (0.977g of BaCl_2_.2H_2_O, 15g of PEG 6000, and 200 µL of Na_2_SO_4_ 50 mM at 100 mL) and 1000 µL of the extract of each sample. Then, the absorbance was measured with a Jenway 6300 Spectrophotometer (Cole-Parmer Instrument Co. Ltd.—Saint Neots, UK) at 660 nm. The quantification took place by measuring 0.12 and 0.52 mm standards made with 10 and 50 µL of Na_2_SO_4_ 50 mM solution and following the same precipitation stage as for the samples (filling up to a final volume of 5 mL with deionized water).

For total S content determination, the principle of the method was the same as the one for sulfates. In the specific determinations, 70 mg of the lyophilized sample was weighed, placed in a crucible, and treated with 5 mL of 7% w/v Mg(NO_3_)_2_ solution. Then, the crucibles were placed on a heated plate (at 200–250 °C) until the solution was completely evaporated, followed by their incineration at 600 °C for 6 h. The ash was then extracted and filtered with 5 mL of 2% *v*/*v* acetic acid. The final dilution of the extracts was 12 mL with acetic acid 2% *v*/*v*. The precipitation and quantification stages were carried out in the exact same ways as for the sulfates.

For chlorophyll and carotenoid determination, 100 mg of lyophilized plant material was supplemented with 7 mL of DMSO solution in a water bath at 65 °C for 15 min. The extracts were filtered and then diluted with DMSO until the final volume of 20 mL. For the determination of the photosynthetic pigments, the absorbance of the extracts was measured at three different wavelengths (470, 646, and 663 nm) with a Jenway 6300 Spectrophotometer (Cole-Parmer Instrument Co., Ltd- Saint Neots, UK). The quantification of each photosynthetic pigment was calculated in mg L^−1^ by the following formulas [62]. Based on the measured weights, the results were converted in mg g^−1^ DM.

Chl*a* (mg L^−1^) = 12.21 × A_663_ − 2.81 × A_646_

Chl*b* (mg L^−1^) = 20.13 × A_646_ − 5.03 × A_663_

Chl_tot_ (mg L^−1^) = Chl*a* + Chl*b*

Car (mg L^−1^) = [1000 × A_470_ − 3.27 × Chl*a* − 104 × Chl*b*]/229

The glucosinolates were extracted and desulfated according to the EU method L170/28 [63], with some modifications. For preparing the raw extract, 50 to 80 mg of the lyophilized sample material was used. The desulfoglucosinolates were measured with a 1260 Infinity HPLC machine (Agilent Technologies Inc.— Santa Clara, CA, USA) with UV detection at 220 nm (by a DAD detector). For the separation of desulfoglucosinolates, 20% *v*/*v* acetonitrile and tridistilled water were used as eluents with a flow rate of 0.8 mL/min. The separation was performed with a Hyperclone C18 ODS column (200 × 4.6 mm, 5 μm, Phenomenex Inc.— Santa Clara, CA, US) and a guard column (Agilent Technologies Inc.— Santa Clara, CA, US) at 30 °C. Finally, the ejection dose was 25 μL and chromatograms were analyzed for the GLS quantification, based on a calibration curve that was produced from standard material (Phytoplan GmbH—Heidelberg, Germany). The determined GSLs were glucoraphanin (GlRa), glucobrassicin (GlBr), and glucoiberin (GlIb) with their retention time being 8.4, 15.2, and 5.2 min, respectively. Additionally, the detected GSLs were also proven using LCMS.

Polyphenols were extracted and analyzed as described by Bloem et al. (2020) [64]. For the extraction, 55 mg of the lyophilized sample material was used and the determination was carried out using a Specord 50 photometer (Analytik Jena GmbH—Jena, Germany). For the quantification, a calibration curve was made with gallic acid as the equivalent.

### 4.3. Statistical Analyses

Two groups of data were made. One contained the different concentrations of sodium selenate solutions and the other included the different amino acid combinations with the 0.2 mM sodium selenate solution. Both groups were analyzed using a two-way analysis of variance (ANOVA) with interaction and the comparison of means was performed using a Tukey–Kramer test at *p* ≤ 0.05. The first factor of the ANOVA was the treatment solution, whilst the second one was the application mode. The R studio software was used for the statistical work and the Microsoft Excel software was used for the table preparation.

## 5. Conclusions

From the studied selenium concentration gradient, the foliar application of Se 0.2 mM (Se0.2/FA,SiE) provided the lowest commercially acceptable Se content per floret (239 μg or 0.3 μmol g^−1^ DM); it reduced S_org_ (−45%), GlIb (−31%), and GlBr (−27%), whilst it increased Car (21%) and GlRa (27%). Coupled with amino acids, the Se 0.2 mM provided commercially acceptable Se contents per floret only via foliar application.

From the studied combinations, Met,Se0.2/FA,IAE provided the lowest Se content per floret (183 μg or 0.2 μmol g^−1^ DM) and increased S_org_ (35%), Car (45%), Chl_tot_ (27%), with no effect on PPs or GSLs. Cys,Met,Se0.2/FA,IAE and mix,Se0.2/FA,IAE increased S_org_ content, too, by 36% and 16%, respectively. Thus, the foliar application with the IAE surfactant was able to increase S_org_, and methionine was the amino acid in common, with varying positive effects on carotenoids and chlorophylls. Only the Cys,Met,Se0.2 combination presented positive effects on GSL, especially GlRa, but it reduced the fresh mass of the floret.

The foliar application with the SiE surfactant failed to positively affect the organic S content. However, in all studied combinations of Se 0.2 mM with amino acids, the Se content per floret was commercially acceptable, whilst it did not affect FM or DM. It also significantly increased the GSL and especially the GlRa and GlIb contents, without affecting PPs, whilst it decreased GlBr except for the one with methionine (Met,Se0.2/FA,SiE). On the other hand, GlBr remained unaffected.

## Figures and Tables

**Table 1:** The performance of the increased selenium concentration (A) or the combination of selenium 0.2 mM with amino acids (B) and application mode on the biofortification of florets. Means +/− SE are provided for (1) fresh mass, (2) dry mass, along with (3) selenium, (4) organic sulfur, (5) sulfate, and (6) total sulfur contents. Different letters indicate statistically significant differences in Tukey–Kramer test at *p* ≤ 0.05. **TR**: statistical evaluation among the application modes. **AppM**: statistical evaluation among the treatments. **Dash** indicates that the two-way ANOVA did not reveal any differences at the *p* < 0.05 level. In the **A** group, control treatment served the fertigation without Se application (Se0.0/FERT). In the **B** group, control treatment served the fertigation with Se 0.2 mM (Se0.2/FERT).

	1. Fresh Mass (g)													2. Dry Mass (g)												
	FERT				FA, IAE				FA, SiE				TR	FERT				FA, IAE				FA, SiE				TR
**A ** **Se 0.0**	77	±	4	-	61	±	6	-	61	±	7	-	AΒ	10.96	±	0.91	-	8.38	±	1.20	-	9.65	±	0.87	-	-
**Se 0.2**	81	±	5	-	77	±	7	-	73	±	3	-	A	10.75	±	0.48	-	12.41	±	0.49	-	10.04	±	0.51	-	-
**Se 1.5**	64	±	2	-	71	±	7	-	61	±	5	-	AΒ	9.62	±	0.58	-	10.49	±	1.36	-	9.29	±	1.07	-	-
**Se 3.0**	54	±	12	-	68	±	3	-	65	±	4	-	Β	11.26	±	0.53	-	9.73	±	0.39	-	11.87	±	2.47	-	-
**AppM**		-				-				-					-				-				-			
**B ** **Se 0.2**	81	±	5	-	77	±	7	-	73	±	3	-	AΒ	10.75	±	0.48	ab	12.41	±	0.49	ab	10.04	±	0.51	ab	AB
**Cys, Se 0.2**	90	±	4	-	91	±	2	-	73	±	5	-	A	13.33	±	0.19	a	12.54	±	0.03	ab	9.87	±	1.05	ab	A
**Met, Se 0.2**	86	±	3	-	81	±	5	-	70	±	4	-	AΒ	11.95	±	0.63	ab	11.53	±	0.69	ab	9.84	±	0.44	ab	AB
**Cys, Met, Se 0.2**	71	±	6	-	63	±	7	-	76	±	6	-	Β	8.99	±	0.47	b	8.56	±	1.14	b	11.57	±	1.17	ab	B
**mix, Se 0.2**	77	±	4	-	62	±	9	-	76	±	12	-	AΒ	10.24	±	0.44	ab	10.23	±	1.55	ab	11.84	±	1.14	ab	AB
**AppM**		-				-				-					-				-				-			
	**3. Selenium content (μmol g^−1^ DM)**													**4. Total sulfur content (μmol g^−1^ DM)**												
	**FERT**				**FA, IAE**				**FA, SiE**				**TR**	**FERT**				**FA, IAE**				**FA, SiE**				**TR**
**A ** **Se 0.0**	0.0	±	0.0	e	0.0	±	0.0	e	0.0	±	0.0	e	C	60.2	±	6.1	-	47.1	±	2.7	-	62.9	±	6.4	-	A
**Se 0.2**	0.7	±	0.1	de	0.5	±	0.1	de	0.3	±	0.0	de	C	54.5	±	3.3	-	59.7	±	4.0	-	39.0	±	3.4	-	AB
**Se 1.5**	4.4	±	0.2	ab	2.0	±	0.2	cd	2.6	±	0.2	bc	B	66.8	±	2.4	-	56.8	±	4.5	-	54.0	±	1.9	-	A
**Se 3.0**	5.7	±	0.8	a	4.1	±	0.7	ab	2.9	±	0.5	bc	A	46.6	±	10.9	-	41.9	±	5.2	-	46.3	±	4.7	-	B
**AppM**		A’				B’				B’					-				-				-			
**B ** **Se 0.2**	0.7	±	0.1	ab	0.5	±	0.1	bc	0.3	±	0.0	c	A	54.5	±	3.3	-	59.7	±	4.0	-	39.0	±	3.4	-	-
**Cys, Se 0.2**	0.8	±	0.1	a	0.3	±	0.1	c	0.4	±	0.1	c	A	50.2	±	4.7	-	64.5	±	8.4	-	39.0	±	3.0	-	-
**Met, Se 0.2**	0.7	±	0.1	ab	0.2	±	0.1	c	0.2	±	0.0	c	A	47.9	±	10.8	-	73.8	±	4.6	-	34.8	±	4.5	-	-
**Cys, Met, Se 0.2**	0.9	±	0.0	a	0.2	±	0.1	c	0.3	±	0.1	c	A	41.4	±	2.1	-	72.8	±	5.2	-	41.5	±	1.8	-	-
**mix, Se 0.2**	0.7	±	0.1	ab	0.2	±	0.0	c	0.3	±	0.0	c	A	54.9	±	9.8	-	63.4	±	11.9	-	45.0	±	2.1	-	-
**AppM**		A’				B’				B’					B’				A’				C’			
	**5. Sulfate content (μmol g^−1^ DM)**													**6. Organic sulfur content (μmol g^−1^ DM)**												
	**FERT**				**FA, IAE**				**FA, SiE**				**TR**	**FERT**				**FA, IAE**				**FA, SiE**				**TR**
**A ** **Se 0.0**	11.3	±	0.7	-	10.2	±	1.3	-	13.2	±	3.2	-	B	48.9	±	5.6	-	36.9	±	2.7	-	49.6	±	3.3	-	A
**Se 0.2**	18.0	±	1.6	-	20.8	±	4.3	-	12.1	±	1.1	-	AB	36.5	±	1.7	-	39.0	±	1.4	-	26.9	±	2.3	-	AB
**Se 1.5**	19.0	±	1.3	-	24.5	±	4.4	-	21.1	±	2.7	-	A	47.8	±	3.6	-	32.4	±	4.2	-	32.9	±	1.8	-	A
**Se 3.0**	18.7	±	2.0	-	25.8	±	2.0	-	19.1	±	3.0	-	A	27.9	±	11.8	-	16.2	±	6.9	-	27.2	±	3.5	-	B
**AppM**		-				-				-					A’				B’				A’B’			
**B ** **Se 0.2**	18.0	±	1.6	-	20.8	±	4.3	-	12.1	±	1.1	-	-	36.5	±	1.7	-	39.0	±	1.4	-	26.9	±	2.3	-	-
**Cys, Se 0.2**	19.2	±	0.5	-	18.2	±	2.7	-	21.4	±	3.6	-	-	31.0	±	4.7	-	46.3	±	9.9	-	17.6	±	2.8	-	-
**Met, Se 0.2**	16.7	±	1.8	-	17.4	±	2.5	-	18.1	±	3.6	-	-	31.2	±	9.1	-	56.4	±	2.8	-	16.7	±	1.2	-	-
**Cys, Met, Se 0.2**	27.6	±	2.5	-	24.4	±	3.0	-	16.8	±	0.3	-	-	13.8	±	1.3	-	48.4	±	6.0	-	24.7	±	2.1	-	-
**mix, Se 0.2**	21.6	±	5.7	-	23.5	±	5.9	-	23.3	±	3.8	-	-	33.3	±	14.1	-	40.0	±	13.2	-	21.7	±	4.4	-	-
**AppM**		-				-				-					B’				A’				B’			

**Table 2:** The performance of the increased selenium concentration (A) or the combination of selenium 0.2 mM with amino acids (B) and application mode on the biofortification of florets. Means +/− SE are provided for (1) carotenoids, (2) total chlorophylls, (3) chlorophyll-a, and (4) chlorophyll-b contents. Different letters indicate statistically significant differences in Tukey–Kramer test at *p* ≤ 0.05. **TR**: statistical evaluation among treatments. **AppM**: statistical evaluation among application modes. **Dash** indicates that the two-way ANOVA did not reveal any differences at the *p* < 0.05 level. In the **A** group, control treatment served the fertigation without Se application (Se0.0/FERT). In the **B** group, control treatment served the fertigation with Se 0.2 mM (Se0.2/FERT).

	1. Carotenoids Content (mg g^−1^ DM)													2. Total Chlorophylls Content (mg g^−1^ DM)												
	FERT				FA, IAE				FA, SiE				TR	FERT				FA, IAE				FA, SiE				TR
**A ** **Se 0.0**	0.14	±	0.02	a	0.17	±	0.01	a	0.13	±	0.01	a	**-**	1.44	±	0.11	a	1.58	±	0.11	a	1.37	±	0.05	a	**-**
**Se 0.2**	0.11	±	0.01	a	0.15	±	0.01	a	0.17	±	0.01	a	**-**	1.19	±	0.07	a	1.54	±	0.06	a	1.40	±	0.07	a	**-**
**Se 1.5**	0.14	±	0.01	a	0.13	±	0.00	a	0.15	±	0.01	a	**-**	1.42	±	0.02	a	1.36	±	0.04	a	1.62	±	0.12	a	**-**
**Se 3.0**	0.14	±	0.01	a	0.12	±	0.02	a	0.16	±	0.01	a	**-**	1.39	±	0.04	a	1.26	±	0.15	a	1.48	±	0.12	a	**-**
**AppM**		-				-				-					-				-				-			
**B ** **Se 0.2**	0.11	±	0.01	**-**	0.15	±	0.01	**-**	0.17	±	0.01	**-**	**-**	1.19	±	0.07	**-**	1.54	±	0.06	**-**	1.40	±	0.07	**-**	**-**
**Cys, Se 0.2**	0.17	±	0.01	**-**	0.13	±	0.03	**-**	0.16	±	0.02	**-**	**-**	1.62	±	0.13	**-**	1.21	±	0.28	**-**	1.52	±	0.16	**-**	**-**
**Met, Se 0.2**	0.14	±	0.02	**-**	0.16	±	0.01	**-**	0.17	±	0.01	**-**	**-**	1.29	±	0.14	**-**	1.51	±	0.06	**-**	1.64	±	0.11	**-**	**-**
**Cys, Met, Se 0.2**	0.18	±	0.02	**-**	0.18	±	0.01	**-**	0.16	±	0.01	**-**	**-**	1.61	±	0.15	**-**	1.71	±	0.08	**-**	1.43	±	0.06	**-**	**-**
**mix, Se 0.2**	0.14	±	0.02	**-**	0.15	±	0.03	**-**	0.16	±	0.01	**-**	**-**	1.38	±	0.13	**-**	1.52	±	0.23	**-**	1.45	±	0.05	**-**	**-**
**AppM**		**-**				**-**				**-**					-				-				-			
	**3. Chlorophyll-a content (mg g^−1^ DM)**													**4. Chlorophyll-b content (mg g^−1^ DM)**												
	**FERT**				**FA, IAE**				**FA, SiE**				**TR**	**FERT**				**FA, IAE**				**FA, SiE**				**TR**
**A ** **Se 0.0**	0.99	±	0.08	a	1.11	±	0.09	a	0.94	±	0.04	a	**-**	0.44	±	0.02	**-**	0.47	±	0.02	**-**	0.43	±	0.01	**-**	**-**
**Se 0.2**	0.80	±	0.06	a	1.06	±	0.04	a	0.98	±	0.06	a	**-**	0.38	±	0.01	**-**	0.48	±	0.02	**-**	0.42	±	0.01	**-**	**-**
**Se 1.5**	0.98	±	0.02	a	0.93	±	0.03	a	1.13	±	0.09	a	**-**	0.44	±	0.00	**-**	0.43	±	0.01	**-**	0.49	±	0.03	**-**	**-**
**Se 3.0**	0.97	±	0.03	a	0.85	±	0.11	a	1.03	±	0.09	a	**-**	0.43	±	0.01	**-**	0.41	±	0.04	**-**	0.45	±	0.04	**-**	**-**
**AppM**		**-**				**-**				**-**					-				-				-			
**B ** **Se 0.2**	0.80	±	0.06	**-**	1.06	±	0.04	**-**	0.98	±	0.06	**-**	**-**	0.38	±	0.01	**-**	0.48	±	0.02	**-**	0.42	±	0.01	**-**	**-**
**Cys, Se 0.2**	1.12	±	0.09	**-**	0.82	±	0.21	**-**	1.05	±	0.12	**-**	**-**	0.50	±	0.04	**-**	0.39	±	0.07	**-**	0.47	±	0.04	**-**	**-**
**Met, Se 0.2**	0.89	±	0.11	**-**	1.04	±	0.05	**-**	1.11	±	0.06	**-**	**-**	0.40	±	0.03	**-**	0.47	±	0.01	**-**	0.53	±	0.05	**-**	**-**
**Cys, Met, Se 0.2**	1.14	±	0.12	**-**	1.23	±	0.07	**-**	0.99	±	0.04	**-**	**-**	0.47	±	0.04	**-**	0.52	±	0.01	**-**	0.44	±	0.01	**-**	**-**
**mix, Se 0.2**	0.96	±	0.10	**-**	1.06	±	0.18	**-**	1.01	±	0.04	**-**	**-**	0.42	±	0.03	**-**	0.46	±	0.06	**-**	0.44	±	0.01	**-**	**-**
**AppM**		-				-				-					-				-				-			

**Table 3:** The performance of the gradient of selenium concentration (A) or the combination of selenium 0.2 mM with amino acids (B) and application mode on the biofortification of florets. Means +/− SE are provided for (1) polyphenols, (2) total glucosinolates, (3) glucoraphanine, (4) glucoiberin, and (5) glucobrassicin contents. Different letters indicate statistically significant differences in Tukey–Kramer test at *p* ≤ 0.05. **TR**: statistical evaluation among treatments. **AppM**: statistical evaluation among application modes. **Dash** indicates that the two-way ANOVA did not reveal any differences at the *p* < 0.05 level. In the **A** group, control treatment served the fertigation without Se application (Se0.0/FERT). In the **B** group as control treatment served the fertigation with Se 0.2 mM (Se0.2/FERT).

	1. Polyphenols (GAE) Content (mg g^−1^ DM)													2. Total glucosinolates Content (mg g^−1^ DM)												
	FERT				FA, IAE				FA, SiE				TR	FERT				FA, IAE				FA, SiE				TR
**A ** **Se 0.0**	8.27	±	0.13	-	7.78	±	0.94	-	7.81	±	0.51	-	B	2.34	±	0.18	-	2.83	±	0.59	-	2.47	±	0.32	-	-
**Se 0.2**	9.87	±	0.28	-	10.62	±	0.37	-	8.81	±	0.50	-	A	2.83	±	0.18	-	2.89	±	0.27	-	2.60	±	0.23	-	-
**Se 1.5**	9.52	±	0.67	-	9.28	±	0.36	-	8.24	±	1.09	-	AB	1.95	±	0.27	-	3.36	±	0.80	-	3.05	±	0.83	-	-
**Se 3.0**	9.75	±	1.08	-	10.03	±	0.25	-	7.82	±	0.25	-	AB	1.89	±	0.10	-	3.22	±	0.17	-	2.73	±	0.01	-	-
**AppM**		A’				A’				B’					B’				A’				A’B’			
**B ** **Se 0.2**	9.87	±	0.28	-	10.62	±	0.37	-	8.81	±	0.50	-	-	2.83	±	0.18	-	2.89	±	0.27	-	2.60	±	0.23	-	B
**Cys, Se 0.2**	10.76	±	0.20	-	10.45	±	0.99	-	9.47	±	0.69	-	-	2.52	±	0.09	-	2.65	±	0.32	-	4.01	±	0.24	-	B
**Met, Se 0.2**	9.81	±	0.74	-	9.89	±	1.07	-	8.54	±	0.22	-	-	3.59	±	0.48	-	2.80	±	0.36	-	4.24	±	0.17	-	AB
**Cys, Met, Se 0.2**	8.65	±	0.71	-	9.57	±	0.70	-	10.01	±	0.65	-	-	3.87	±	0.64	-	3.89	±	0.96	-	4.35	±	0.20	-	A
**mix, Se 0.2**	9.15	±	0.26	-	10.14	±	0.33	-	9.55	±	0.86	-	-	3.91	±	0.12	-	2.45	±	0.09	-	4.30	±	0.26	-	AB
**AppM**		-				-				-					A’B’				B’				A’			
	**3. Glucoraphanin content (mg g^−1^ DM)**													**4. Glucoiberin content (mg g^−1^ DM)**												
	**FERT**				**FA, IAE**				**FA, SiE**				**TR**	**FERT**				**FA, IAE**				**FA, SiE**				**TR**
**A ** **Se 0.0**	1.64	±	0.14	-	2.21	±	0.53	-	1.85	±	0.22	-	-	0.13	±	0.01	abc	0.15	±	0.02	abc	0.14	±	0.01	abc	-
**Se 0.2**	2.16	±	0.15	-	2.06	±	0.26	-	2.09	±	0.23	-	-	0.18	±	0.00	ab	0.22	±	0.02	a	0.09	±	0.02	bc	-
**Se 1.5**	1.48	±	0.21	-	2.59	±	0.75	-	2.54	±	0.79	-	-	0.12	±	0.02	abc	0.20	±	0.06	ab	0.15	±	0.01	abc	-
**Se 3.0**	1.41	±	0.08	-	2.53	±	0.12	-	2.26	±	0.02	-	-	0.11	±	0.01	abc	0.21	±	0.00	a	0.06	±	0.02	c	-
**AppM**		B’				A’				A’B’					B’				A’				B’			
**B ** **Se 0.2**	2.16	±	0.15	-	2.06	±	0.26	-	2.09	±	0.23	-	B	0.18	±	0.00	bcd	0.22	±	0.02	bcd	0.09	±	0.02	d	B
**Cys, Se 0.2**	2.00	±	0.10	-	1.94	±	0.22	-	3.22	±	0.23	-	B	0.18	±	0.02	bcd	0.16	±	0.01	bcd	0.28	±	0.01	abc	AB
**Met, Se 0.2**	2.93	±	0.44	-	2.17	±	0.32	-	3.54	±	0.17	-	AB	0.25	±	0.05	abcd	0.18	±	0.01	bcd	0.29	±	0.01	abc	A
**Cys, Met, Se 0.2**	3.06	±	0.51	-	3.32	±	0.93	-	3.62	±	0.20	-	A	0.24	±	0.03	abcd	0.18	±	0.08	bcd	0.32	±	0.03	ab	A
**mix, Se 0.2**	3.14	±	0.12	-	1.84	±	0.08	-	3.55	±	0.21	-	AB	0.24	±	0.01	abcd	0.14	±	0.02	cd	0.39	±	0.05	a	A
**AppM**		B’				B’				A’					B’				B’				A’			

			**5. Glucobrassicin content (mg g^−1^ DM)**																							
			**FERT**								**FA, IAE**						**FA, SiE**							**TR**		
**A** **Se 0.0**			0.56			±	0.07		-		0.47		±	0.05		-	0.48			±	0.09		-	-		
**Se 0.2**			0.49			±	0.04		-		0.62		±	0.03		-	0.41			±	0.03		-	-		
**Se 1.5**			0.36			±	0.05		-		0.57		±	0.02		-	0.36			±	0.03		-	-		
**Se 3.0**			0.38			±	0.01		-		0.48		±	0.05		-	0.40			±	0.01		-	-		
**AppM**						B’							A’							B’						
**B** **Se 0.2**			0.49			±	0.04		ab		0.62		±	0.03		a	0.41			±	0.03		ab	-		
**Cys, Se 0.2**			0.34			±	0.03		b		0.54		±	0.10		ab	0.51			±	0.02		ab	-		
**Met, Se 0.2**			0.41			±	0.01		ab		0.46		±	0.04		ab	0.42			±	0.03		ab	-		
**Cys, Met, Se 0.2**			0.57			±	0.10		ab		0.39		±	0.01		ab	0.40			±	0.03		ab	-		
**mix, Se 0.2**			0.54			±	0.06		ab		0.46		±	0.01		ab	0.35			±	0.07		b	-		
**AppM**						-							-							-						

**Table 4:** Overview of the performance of the application mode per treatment for the applied Se concentrations, in terms of percentage difference with the control plants (Se0.0/FERT). The percentage of changes was evaluated against the selenium content of florets either as μmol g^−1^ DM or μg floret^−1^.

	**Se**	**Se**	**FM**	**DM**	**Sorg**	**SO4-S**	**Stot**	**Car**	**Chltot**	**Chla**	**Chlb**	**PP**	**ΣGSL**	**GlRa**	**GlIb**	**GlBr**
**Se0.0/FERT**	0	0	77	11	48.9	11.3	60.2	0.14	1.44	0.99	0.44	8.27	2.34	1.64	0.13	0.56
	μmol g^−1^ DM	μg floret^−1^	g		μmol g^−1^ DM			mg g^−1^ DM								
**Treatment**			**Application mode**													
			Δ(%) compared with Se0.0/FERT as control													
			**Fertigation**													
**Se 0.2**	0.7	597			**−25**	**59**		**−21**	**−17**	**−19**		**19**	**21**	**32**	**38**	
**Se 1.5**	4.4	3356	**−17**			**68**						**15**	**−17**			**−36**
**Se 3.0**	5.7	5089	**−30**		**−43**	**65**	**−23**					**18**	**−19**		**−15**	**−32**
			**Foliar application plus IAE**													
**Se0.0/FA,IAE**	0	0	**−21**	**−24**	**−25**	**−10**	**−22**	**21**				**-6**	**21**	**35**		**−16**
**Se 0.2**	0.5	492			**−20**	**84**						**28**	**24**	**26**	**69**	
**Se 1.5**	2.0	1663			**−34**	**117**							**44**	**58**	**54**	
**Se 3.0**	4.1	3163			**−67**	**128**	**−30**					**21**	**38**	**54**	**62**	
			**Foliar application plus SiE**													
**Se0.0/FA,SiE**	0	0	**−21**	**−12**		**17**										
**Se 0.2**	0.3	239			**−45**		**−35**	**21**						**27**	**−31**	**−27**
**Se 1.5**	2.6	1915	**−21**	**−15**	**−33**	**87**							**30**	**55**		**−36**
**Se 3.0**	2.9	2729	**−16**		**−44**	**69**	**−23**						**17**	**38**	**−54**	**−29**

**Table 5:** Overview of the performance of the application mode per treatment for the applied Se 0.2 mM concentration enriched with cysteine, methionine, cysteine plus methionine, or the mix of phenylalanine, tryptophane, and methionine, in terms of percentage difference with the control plants (Se0.2/FERT). The percentage of changes was evaluated against the selenium content of florets either as μmol g^−1^ DM or μg floret^−1^.

	**Se**	**Se**	**FM**	**DM**	**Stot**	**SO4-S**	**Sorg**	**Car**	**Chltot**	**Chla**	**Chlb**	**PP**	**ΣGSL**	**GlRa**	**GlIb**	**GlBr**
**Se 0.2/FERT**	0.7	597	81	11	54.5	18	37	0.11	1.19	0.8	0.38	9.9	2.83	2.16	0.18	0.49
	μmol g^−1^ DM	μg floret^−1^	g		μmol g^−1^ DM			mg g^−1^ DM								
**Treatment**			**Application mode**													
			Δ(%) compared with Se 0.2 /FERT as control													
			**Fertigation**													
**Cys, Se 0.2**	0.8	846		**24**				**55**	**36**	**40**	**32**					**− 31**
**Met, Se 0.2**	0.7	663						**27**					**27**	**36**	**39**	**−16**
**Cys, Met, Se 0.2**	0.9	642			**−24**	**53**	**−62**	**64**	**35**	**43**	**24**		**37**	**42**	**33**	**16**
**mix, Se 0.2**	0.7	568				**20**		**27**	**16**	**20**			**38**	**45**	**33**	
			**Foliar application plus IAE**													
**Se 0.2/FA, IAE**	0.4	492				**16**		**36**	**29**	**33**	**26**				**22**	**27**
**Cys, Se 0.2**	0.3	298		**17**	**18**		**27**	**18**								
**Met, Se 0.2**	0.2	183			**35**		**55**	**45**								
**Cys, Met, Se 0.2**	0.2	136	**− 22**	**− 20**	**34**	**36**	**33**	**64**	**47**	**54**	**37**		**37**	**54**		**− 20**
**mix, Se 0.2**	0.2	162	**− 23**		**16**	**31**		**36**	**28**	**33**	**21**				**− 22**	
		162	**Foliar application plus SiE**													
**Se 0.2/FA,SiE**	0.3	239			**− 28**	**− 33**	**− 26**	**55**	**18**	**23**					**−50**	**− 16**
**Cys, Se 0.2**	0.4	313			**− 28**	**19**	**− 52**	**45**	**28**	**31**	**24**		**42**	**49**	**56**	
**Met, Se 0.2**	0.2	156			**− 36**		**− 54**	**55**	**38**	**39**	**39**		**50**	**64**	**61**	
**Cys, Met, Se 0.2**	0.3	275			**− 24**		**− 32**	**45**	**20**	**24**	**16**		**54**	**68**	**78**	**− 18**
**mix, Se 0.2**	0.3	282			**− 17**	**29**	**− 41**	**45**	**22**	**26**	**16**		**52**	**64**	**117**	**− 29**

## Data Availability

The data presented in this study are available on request from the corresponding author. The data are not publicly available due to privacy.

## Abbreviations

Se	Selenium	FM	Fresh mass
Se0.2	Selenium concentration 0.2 mM	DM	Dry mass
GSLs	Glucosinolates	C	Carbon
ΣGSL	Total determined glucosinolates	N	Nitrogen
GlRa	Glucoraphanin	S	Sulfur
GlIb	Glucoiberin	SO4-S	Sulfate
GlBr	Glucobrassicin	S_tot_	Total sulfur
FERT	Fertigation	S_org_	Organic sulfur
FA	Foliar application	Car	Carotenoids
IAE	Isodecyl alcohol ethoxylate	Chl_tot_	Total chlorophyll
SiE	Organosilicon-based ethoxylate	Chla	Chlorophyll-a
FA,SiE	Foliar application with SiE	Chlb	Chlorophyll-b
FA,IAE	Foliar application with IAE	PPs	Polyphenols
Cys	Cysteine	Met	Methionine
Mix	Mixture of phenylalanine, tryptophane, and methionine		
